# Factors associated with the utilisation of skilled delivery services in Papua New Guinea: evidence from the 2016–2018 Demographic and Health Survey

**DOI:** 10.1093/inthealth/ihab007

**Published:** 2021-03-03

**Authors:** Abdul-Aziz Seidu, Bright Opoku Ahinkorah, Ebenezer Agbaglo, Joseph Kojo Oduro, Abigail Amoah, Sanni Yaya

**Affiliations:** Department of Population and Health, Faculty of Social Sciences, College of Humanities and Legal Studies, University of Cape Coast, Cape Coast, Ghana; College of Public Health, Medical and Vertinary Sciences, James Cook University, Townsville, QLD 4811, Australia; School of Public Health, Faculty of Health, University of Technology Sydney, Sydney, NSW 2007, Australia; Department of English, University of Cape Coast, Cape Coast, Ghana; Department of Population and Health, Faculty of Social Sciences, College of Humanities and Legal Studies, University of Cape Coast, Cape Coast, Ghana; Department of Population and Health, Faculty of Social Sciences, College of Humanities and Legal Studies, University of Cape Coast, Cape Coast, Ghana; School of International Development and Global Studies, University of Ottawa, Ottawa, ON KIN 6N5, Canada; The George Institute for Global Health, Imperial College London, London W120BZ, UK

**Keywords:** demographic and health surveys, global health, Papua New Guinea, skilled delivery, women

## Abstract

**Background:**

We sought to determine the prevalence and factors associated with the use of skilled assistance during delivery in Papua New Guinea.

**Methods:**

We analysed nationally representative data from 5210 women in Papua New Guinea using the 2016–2018 Demographic and Health survey. Both bivariate and multivariable analyses were performed. Statistical significance was set at p<0.05.

**Results:**

The prevalence of skilled assistance during delivery was 57.6%. The richest women (adjusted OR [AOR]=3.503, 95% CI 2.477 to 4.954), working women (AOR=1.221, 95% CI 1.037 to 1.439), women with primary (AOR=1.342, 95% CI 1.099 to 1.639), secondary or higher education (AOR=2.030, 95% CI 1.529 to 2.695), women whose partners had a secondary or higher level of education (AOR=1.712, 95% CI 1.343 to 2.181], women who indicated distance was not a big problem in terms of healthcare (AOR=1.424, 95% CI 1.181 to 1.718), women who had ≥4 antenatal care (ANC) visits (AOR=10.63, 95% CI 8.608 to 13.140), women from the Islands region (AOR=1.305, 95% CI 1.045 to 1.628), those who read newspapers or magazines (AOR=1.310, 95% CI 1.027 to 1.669) and women who watched television (AOR=1.477, 95% CI 1.054 to 2.069) less than once a week had higher odds of utilising skilled attendants during delivery. On the contrary, women in the Momase region (AOR=0.543, 95% CI 0.438 to 0.672), women in rural areas (AOR=0.409, 95% CI 0.306 to 0.546), as well as women with a parity of 3 (AOR=0.666, 95% CI 0.505 to 0.878) or ≥4 (AOR=0.645, 95% CI 0.490 to 0.850) had lower odds of utilising skilled attendance during delivery.

**Conclusion:**

There is relatively low use of skilled delivery services in Papua New Guinea. Wealth, employment status, educational level, parity and number of ANC visits, as well as access to healthcare and place of residence, influence the utilisation of skilled delivery services.

## Introduction

Skilled birth attendants (SBAs), such as doctors, nurses, midwives or trained village health workers, are people with expertise in midwifery.^1^ With such expertise, SBAs, through their services, have the capacity to reduce maternal mortality, as stipulated in the UN's Sustainable Development Goal 3.1 (SDG 3.1), and also ensure child survival.^2,3^ However, research suggests that developing countries record a low use of services provided by SBAs.^4^ This has resulted in a higher prevalence of disease and deaths related to maternity in developing countries, most of which result from otherwise preventable causes such as obstructed labour, sepsis, haemorrhage and eclampsia.^5^ For instance, the United Nations^6^ reports that, as of 2013, developing countries recorded about 14 times the maternal mortality recorded in high-income countries. A similar report indicated that, as of 2015, 99% of maternal deaths recorded worldwide occurred in developing countries.^7^

With the current situation on the ground, it becomes imperative to explore the factors associated with SBA utilisation in low- and middle-income countries and several studies have been conducted in this regard. For example, Olakunde et al.,^8^ focusing on married adolescents, investigated the factors associated with SBA uptake in Nigeria. Also, focusing specifically on rural Nigeria, Solanke and Rahman^9^ explored factors associated with SBA uptake during delivery. Other countries that have featured in studies of this kind include Ghana,^10,11^ Ethiopia^12,13^ and Kenya.^14^ Generally, such studies have revealed some associations between SBA uptake and various socioeconomic variables. It has, for instance, been revealed that people with educated family members, those whose best friends use maternal care^13^ and those with access to mass media^9^ are highly likely to use services provided by SBAs.

As a way of contributing to the growing literature on SBA, the present study investigates factors associated with the uptake of SBAs in Papua New Guinea. This study is important for two main reasons. In the first place, the present study extends studies of this kind in terms of geography, as Papua New Guinea has not featured in any study of this kind. More importantly, research suggests that, in Papua New Guinea, huge numbers of woman deliver at home, without the services of SBAs, which results in high maternal mortality of about 733 maternal deaths per 100 000 live births.^15,16^ Therefore, it is important to reveal the factors that influence the use of SBAs in Papua New Guinea, as this will inform some policy interventions aimed at reducing maternal mortality in the country of focus to help attain SDG 3.1.

## Methods

### Data and sampling design

This study analysed data from the 2016–2018 Papua New Guinea Demographic and Health Survey (PNGDHS), which were collected from October 2016 to December 2018. Among the aims of the PNGDHS is to give current information on basic demographic and health pointers. The survey specifically gathered information on fertility, awareness and the use of family planning methods, breastfeeding practices, the nutritional status of children, maternal and child health, childhood immunisation, adult and childhood mortality, women's empowerment, domestic violence, malaria, awareness and behaviour regarding HIV/AIDS and other sexually transmitted infections, as well as other health-related issues. Technical assistance for the survey was offered by inner city fund (ICF) through the Demographic and Health Survey Programme. Financial assistance was given by the Government of Papua New Guinea, the Australian Government Department of Foreign Affairs and Trade, the United Nations Population Fund (UNFPA) and UNICEF.^17^ The survey used the list of census units (CUs) from the 2011 Papua New Guinea National Population and Housing Census as the sampling frame.

The survey adopted a two-stage stratified sampling technique. The provinces in the country of focus were further divided into 43 strata, paying attention to urban-rural differentials; however, the National Capital District did not have any rural strata. Each stratum provided samples of CUs, and this was done independently in two stages. The first stage involved the use of probability proportional-to-size sampling. The second stage of sampling involved the selection of 24 households from each of the clusters, using an equal probability systematic selection, with the resulting sample consisting of about 19 200 households. During the survey, the enumerators were able to cover 16 745 out of the 17 505; 16 021 of the occupied households were interviewed, with a response rate of 96%; 18 175 women of reproductive age were identified in the interviewed households for individual interviews, with 15 198 women completing the interviews at a response rate of 84%. The sample for the present study comprised 5210 women who had given birth to live babies within the 3 y prior to the survey. We realised that some women had given birth to more than one live birth during the selected period; in such cases, we only focused on the most recent birth. Details of the methodology, pretesting, training of field workers, the sampling design and selection are available in the PNGDHS final report, which is available at https://dhsprogram.com/publications/publication-fr364-dhs-final-reports.cfm. The dataset can be accessed at https://dhsprogram.com/data/dataset/Papua-New-Guinea_Standard-DHS_2017.cfm?flag=0.

### Variables

#### Dependent variable

The binary response—whether or not a woman had given birth with the assistance of an SBA—was considered to be the outcome variable.^17^ From the PNGDHS, a skilled attendant delivery is a birth delivered with the assistance of doctors, midwives, nurses (including trained community health workers) or trained village health volunteers (p. 138).^17^ In this study, skilled delivery, supervised delivery and skilled provider at birth are used interchangeably.

#### Independent variables

Seventeen explanatory variables were considered in this study, based on their availability in the dataset^17^ and conclusions drawn from them associated with skilled delivery in previous studies.^9,10,13^,^18–21^ The variables comprised maternal age, wealth, working status, education, partner's education, marital status, place of residence, region of residence, parity (birth order), getting money for treatment, distance to health facility, antenatal care (ANC) attendance, exposure to mass media (radio, television, newspapers) and gender of the head of the household. Some of these variables were recoded. ANC attendance was recoded into 0, 1, 2, 3 and ≥4 visits. Parity was categorised as 1, 2, 3 or ≥4 births. Education and partner's education were classified into three categories: no education, primary education and secondary education/higher education. Occupation was captured as working or not working and the decision-maker on healthcare was captured as either alone or not alone (Table 1).

**Table 1.** Background characteristics and uptake of skilled delivery services among women in Papua New Guinea

	N=5210		Skilled delivery	
Variable	Frequency	%	No (%)	Yes (%)
Age, y (χ^2^=49.9, p<0.001)				
15–19	181	3.5	30.91	69.09
20–24	1116	21.4	33.49	66.51
25–29	1403	26.9	35.85	64.15
30–34	1098	21.1	35.01	64.99
35–39	853	16.4	42.61	57.39
40–44	412	7.9	36.73	63.27
45–49	149	2.9	58.04	41.96
Wealth (χ^2^=1000.3, p<0.001)				
Poorest	1117	21.4	69.00	31.00
Poorer	1055	20.3	57.34	42.66
Middle	1041	20.0	41.42	58.58
Richer	1020	19.6	24.39	75.61
Richest	977	18.8	7.45	92.55
Working (χ^2^=70.4, p<0.001)				
Not working	3480	66.8	41.09	58.91
Working	1730	33.2	29.45	70.55
Education (χ^2^=837.2, p<0.001)				
No education	1358	26.1	66.73	33.27
Primary	2543	48.8	38.67	61.33
Secondary/higher	1309	25.1	11.93	88.07
Partner's education (χ^2^=668.3, p<0.001)				
No education	1097	21.1	63.52	36.48
Primary	2235	42.9	44.28	55.72
Secondary/higher	1878	36.1	17.05	82.95
Marital status (χ^2^=5.6, p<0.05)				
Married	4281	82.2	36.11	63.89
Cohabiting	929	17.8	40.29	59.71
Region (χ^2^=332.3, p<0.001)				
Southern region	1010	19.4	31.47	68.53
Highlands region	1991	38.2	42.57	57.43
Momase region	1490	28.6	54.22	45.78
Islands region	720	13.8	20.05	79.95
Residence (χ^2^=511.1, p<0.001)				
Urban	562	10.8	8.56	91.44
Rural	4648	89.2	44.91	55.09
Parity (χ^2^=149.6, p<0.001)				
1	1118	21.5	25.97	74.03
2	1042	20.0	29.11	70.89
3	962	18.5	39.19	60.81
≥4	2089	40.1	45.38	54.62
Distance to health facility (χ^2^=550.0, p<0.001)				
Big problem	3132	60.1	51.05	48.95
Not a big problem	2078	39.9	19.56	80.44
Getting money needed for treatment (χ^2^=304.0, p<0.001)				
Big problem	3368	64.6	45.96	54.04
Not a big problem	1842	35.4	21.96	78.04
Decision-maker on healthcare (χ^2^=16.0, p<0.001)				
Not alone	3694	70.9	38.48	61.52
Alone	1516	29.1	32.50	67.50
ANC attendance (χ^2^=1400.1, p<0.001)				
0	1226	23.5	84.53	15.47
1	199	3.8	51.08	48.92
2	355	6.8	39.34	60.66
3	753	14.5	30.39	69.61
≥4	2676	51.4	19.68	80.32
Frequency of reading newspapers or magazines (χ^2^=606.1, p<0.001)				
Not at all	3489	67.0	49.21	50.79
Less than once a week	973	18.7	19.05	80.95
At least once a week	747	14.3	10.99	89.01
Frequency of listening to radio (χ^2^=347.5, p<0.001)				
Not at all	3471	66.6	45.82	54.18
Less than once a week	936	18.0	23.85	76.15
At least once a week	804	15.4	16.26	83.74
Frequency of watching television (χ^2^=391.9, p<0.001)				
Not at all	4133	79.3	43.92	56.08
Less than once a week	458	8.8	15.64	84.36
At least once a week	620	11.9	10.07	89.93
Gender of head of household (χ^2^=36.2, p<0.001)				
Male	4539	87.1	38.38	61.62
Female	671	12.9	26.38	73.62

Abbreviation: ANC, antenatal care.

Source: 2016–2018 PNG DHS.

### Statistical analyses

Three key steps were followed to analyse the data. First, descriptive statistics, such as frequency with %, were executed to represent the background characteristics of study participants and the prevalence of skilled delivery services utilisation. Second, a bivariate analysis using χ^2^ was employed to select candidate variables for the regression analysis. Variables with p<0.05 were moved to the regression analysis stage. At the regression stage, crude and adjusted models were employed. The crude model was estimated to examine the effect of each independent variable on the outcome variable, while multivariable logistic regression was used to examine the effect of all the significant independent variables at the crude level on utilisation of skilled services during delivery. The output was reported as crude ORs (CORs) and adjusted ORs (AORs) with their corresponding 95% CIs. Using the variance inflation factor (VIF), the multicollinearity test showed that there was no evidence of collinearity among the independent variables (mean VIF=1.5, max. VIF=1.99, min. VIF=1.02). We applied sample weight to correct for oversampling and undersampling to ensure generalisation of the findings. The survey (svy) command was applied to take care of the complex sampling procedure involved in the demographic and health surveys. In other words, the svy command was used to declare the data survey data. We carried out the analyses with stata version 14.2 for MacOS (Stata Corporation, College Station, TX, USA).

## Results

### Demographic characteristics of women

Table 1 presents the demographic characteristics of the women interviewed. It was shown that 26.9% of the sample were aged 25–29 y. Regarding wealth, 21.4% were in the poorest wealth quintile. Approximately 69% were not working, 48.8% had a primary level of education, 82.2% were married and 40.1% had ≥4 children.

### Prevalence of skilled delivery in Papua New Guinea

**Figure 1.**
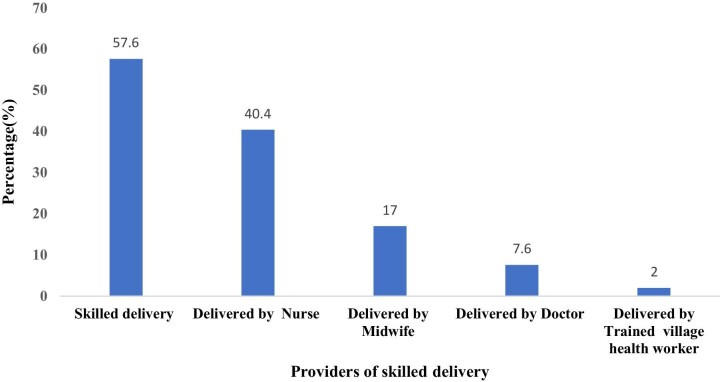
Type of birth attendant during most recent birth among women in Papua New Guinea. Source: 2016–2018 PNGDHS

It was found that 57.6% of the study participants had utilised a skilled delivery service. Among the women who had utilised skilled delivery, 40.4% were attended to by a nurse. Approximately 7 out of 10 women’s deliveries were supervised by skilled personnel (69.1%). Only 2% of them were attended to by a trained village health worker (Figure 1). As Table 1 reveals, a majority of women in the richest wealth quintile (92.6%), 70.6% of those working, 88.1% of those with a secondary or higher level of education, as well as 83.0% of those with educated husbands, 91.4% of those living in urban areas and 74.0% of those with parity had their deliveries supervised by skilled personnel. At the bivariate level, age (χ^2^=49.9, p<0.001), wealth (χ^2^=1000.3, p<0.001), working status (χ^2^=70.4, p<0.001), education (χ^2^=837.2, p<0.001), partner's education (χ^2^=668.3, p<0.001), marital status (χ^2^=5.6, p<0.05), place of residence (χ^2^=511.1, p<0.001), region of residence (χ^2^=332.3, p<0.001), parity (χ^2^=149.6, p<0.001), getting money for treatment (χ^2^=304.0, p<0.001), distance to health facility (χ^2^=550.0, p<0.001), ANC attendance (χ^2^=1400.1, p<0.001), exposure to radio (χ^2^=347.5, p<0.001), television (χ^2^=391.9, p<0.001) and newspapers (χ^2^=606.1, p<0.001), as well as the gender of the head of the household (χ^2^=36.2, p<0.001), were all associated with skilled attendants during delivery at a 95% level of significance (Table 1).

### Multivariable analysis of the determinants of skilled delivery in Papua New Guinea

The results from the study of the factors associated with the use of skilled delivery services in Papua New Guinea are presented. Compared with the poorest women, middle, richer and richest women were, respectively, 1.5 times (AOR=1.452, 95% CI 1.149 to 1.835), 1.9 times (AOR=1.887, 95% CI 1.464 to 2.433) and 3.5 times (AOR=3.503, 95% CI 2.477 to 4.954] more likely to have utilised skilled delivery during their most recent birth. Women who were working (AOR=1.221, 95% CI 1.037 to 1.439] had 1.2 higher odds of utilising skilled providers during delivery compared with those not working. Concerning education, respondents’ and their partners’ levels of education were significantly associated with skilled providers during delivery. For example, compared with mothers with no formal education, those who had completed primary (AOR=1.342, 95% CI 1.099 to 1.639) and secondary or higher (AOR=2.030, 95% CI 1.529 to 2.695) had higher odds (AOR=1.712, 95% CI 1.343 to 2.181) of using skilled providers during delivery. Women whose partners had a secondary or higher level of education had higher odds of utilising skilled providers during delivery compared with those women whose partners had no formal education. In terms of regional variations, compared with those in the southern region, women in the Momase region (AOR=0.543, 95% CI 0.438 to 0.672) had lower odds of skilled delivery while those in the Islands region had higher odds (AOR=1.305, 95% CI 1.045 to 1.628). Relatedly, women in rural areas had lower odds of skilled delivery compared with those in urban centres (AOR=0.409, 95% CI 0.306 to 0.546). In terms of parity, women with a parity of 3 (AOR=0.666, 95% CI 0.505 to 0.878) and ≥4 (AOR=0.645, 95% CI 0.490 to 0.850) had lower odds of skilled delivery compared with those women with a parity of 1. Women who indicated that distance was not a big problem in terms of healthcare had higher odds (AOR=1.424, 95% CI 1.181 to 1.718) compared with those women who indicated that distance was a big problem. The odds of skilled delivery use increased with the number of ANC visits. Specifically, women who had ≥4 ANC visits had about 10.6 times higher odds of utilising skilled providers during delivery compared with those who never attended ANC. Those who read newspapers or magazines (AOR=1.310, 95% CI 1.027 to 1.669) and watched television (AOR=1.477, 95% CI 1.054 to 2.069) less than once a week had higher odds of utilising skilled attendance during delivery compared with those who did not watch television or read no newspapers or magazines at all (Table 2).

**Table 2.** Multivariable analysis of the determinants of skilled delivery services use in Papua New Guinea

Variables	COR [95% CI]	AOR [95% CI]
Age, y		
15–19	Ref	Ref
20–24	0.888 [0.623 to 1.266]	0.833 [0.548 to 1.267]
25–29	0.800 [0.565 to 1.134]	0.942 [0.609 to 1.457]
30–34	0.830 [0.584 to 1.180]	1.001 [0.633 to 1.583]
35–39	0.603** [0.422 to 0.861]	0.911 [0.567 to 1.462]
40–44	0.771 [0.524 to 1.133]	1.163 [0.698 to 1.939]
45–49	0.323*** [0.202 to 0.517]	0.895 [0.463 to 1.730]
Wealth		
Poorest	Ref	Ref
Poorer	1.656*** [1.363 to 2.012]	1.020 [0.812 to 1.281]
Middle	3.147*** [2.601 to 3.808]	1.452** [1.149 to 1.835]
Richer	6.899*** [5.689 to 8.366]	1.887*** [1.464 to 2.433]
Richest	27.66*** [21.40 to 35.75]	3.503*** [2.477 to 4.954]
Working		
Not working	Ref	Ref
Working	1.671*** [1.481 to 1.885]	1.221* [1.037 to 1.439]
Education		
No education	Ref	Ref
Primary	3.180*** [2.743 to 3.687]	1.342** [1.099 to 1.639]
Secondary/higher	14.80*** [12.13 to 18.05]	2.030*** [1.529 to 2.695]
Partner's education		
No education	Ref	Ref
Primary	2.191*** [1.867 to 2.572]	1.148 [0.928 to 1.419]
Secondary/higher	8.473*** [7.083 to 10.14]	1.712*** [1.343 to 2.181]
Marital status		
Married	Ref	Ref
Cohabiting	0.838* [0.723 to 0.970]	0.986 [0.805 to 1.207]
Region		
Southern region	Ref	Ref
Highlands region	0.619*** [0.532 to 0.722]	1.046 [0.845 to 1.295]
Momase region	0.388*** [0.331 to 0.454]	0.543*** [0.438 to 0.672]
Islands region	1.831*** [1.531 to 2.190]	1.305* [1.045 to 1.628]
Residence		
Urban	Ref	Ref
Rural	0.115*** [0.0926 to 0.142]	0.409*** [0.306 to 0.546]
Parity		
1	Ref	Ref
2	0.854 [0.707 to 1.032]	0.938 [0.732 to 1.203]
3	0.544*** [0.451 to 0.657]	0.666** [0.505 to 0.878]
≥4	0.422*** [0.359 to 0.497]	0.645** [0.490 to 0.850]
Distance to health facility		
Big problem	Ref	Ref
Not a big problem	4.289*** [3.783 to 4.863]	1.424*** [1.181 to 1.718]
Getting money needed for treatment		
Big problem	Ref	Ref
Not a big problem	3.022*** [2.662 to 3.431]	1.038 [0.858 to 1.255]
Decision-maker on healthcare		
Not alone	Ref	Ref
Alone	1.299*** [1.143 to 1.40]	1.041 [0.879 to 1.234]
ANC attendance		
0	Ref	Ref
1	5.234*** [3.756 to 7.293]	3.953*** [2.716 to 5.751]
2	8.425*** [6.398 to 11.09]	5.375*** [3.945 to 7.324]
3	12.51*** [9.927 to 15.78]	7.747*** [5.983 to 10.030]
≥4	22.30*** [18.46 to 26.94]	10.63*** [8.608 to 13.140]
Frequency of reading a newspaper or magazine		
Not at all	Ref	Ref
Less than once a week	4.117*** [3.480 to 4.871]	1.310* [1.027 to 1.669]
At least once a week	7.845*** [6.265 to 9.823]	1.235 [0.904 to 1.687]
Frequency of listening to radio		
Not at all	Ref	Ref
Less than once a week	2.701*** [2.290 to 3.186]	0.875 [0.687 to 1.115]
At least once a week	4.357*** [3.602 to 5.270]	1.155 [0.866 to 1.541]
Frequency of watching television		
Not at all	Ref	Ref
Less than once a week	4.225*** [3.282 to 5.439]	1.477* [1.054 to 2.069]
At least once a week	6.992*** [5.410 to 9.037]	0.938 [0.653 to 1.349]
Gender of head of household		
Male	Ref	Ref
Female	1.738*** [1.449 to 2.085]	1.125 [0.879 to 1.439]
N		5210
R^2^		0.270

Abbreviations: ANC, antenatal care; AOR, adjusted OR; COR, crude OR; Ref, reference.

Exponentiated coefficients: 95% CIs in square brackets.

*p<0.05; **p<0.01; ***p<0.001.

Source: 2016–2018 PNG DHS.

### Discussion

The services of SBAs are needed not only during pregnancy but also during childbirth and the postpartum period, as such services help to improve maternal and neonatal survival. The present study assessed the prevalence and factors associated with the utilisation of SBA services in Papua New Guinea. The study reveals that 57.6% of childbearing women in Papua New Guinea utilised skilled assistance during delivery. A similar prevalence of skilled delivery was reported in the PNGDHS 2016–2018, where 56.5% of childbearing women had a ‘skilled provider’ at the birth.^17^ Previous studies in the country by O'keefe et al.^22^ and Mola and Kirby^23^ reported SBA usage of 28% and 39.1%, respectively. One of the possible reasons for the increased prevalence of skilled delivery in the country could be an improved skilled workforce in health facilities. For example, since 2009, the quality of midwifery training in Papua New Guinea has experienced a dramatic improvement and the number of practising midwives has almost tripled, helping to curb the staff shortage the country had been experiencing.^24^ Kep et al.^25^ found that midwives in the country offer some training to birth attendants who work in villages, so as to help increase skilled assistance during delivery. Another possible reason for this increase in SBA uptake could be that pregnant women are sensitised on the need for SBA services during delivery. Notwithstanding, there is still a need to encourage more pregnant women in the country to utilise SBAs during delivery.

We also found that high socioeconomic status (wealth, education and employment) increased the chances of SBA usage during delivery. Specifically, women with a higher wealth status, those with higher levels of education, those whose partners had higher levels of education and those who were employed had a higher likelihood of SBA uptake. Similar studies using nationally representative data in other countries also found that high socioeconomic status increases the likelihood of skilled attendance during delivery.^14,20,21^ Women with higher wealth status and employment are highly likely to use SBA services during delivery, because such women often have the financial empowerment to access skilled attendance during delivery.^26,27^ Such women could be in a better position to pay for transportation costs and the cost of skilled delivery services, if required, due to their financial position. The link between a higher level of education and use of skilled attendance during delivery is not surprising, given that with higher education, women are more likely to be conscious of their health.^20^ Such women are more likely to understand the need for SBA use. This also explains the association between access to mass media and SBA uptake, as revealed by the present study.

Place of residence was found to influence the likelihood of SBA uptake, with rural dwellers, especially residents of the Momase region, having lower odds of skilled delivery compared with urban dwellers. Similar revelations were reported in previous studies.^9,26,28^ This finding could be attributed to the lack of advanced health facilities with highly qualified personnel in the rural areas, which hinders access to skilled health providers for rural women.^29,30^ The health workforce in the country is also characterised by an ageing workforce, with few midwives and community health workers, who are not even given any attractive remuneration to motivate them.^30^ This finding can also be explained in terms of the socioeconomic situation in rural areas, often characterised by low employment, low income levels and low education levels,^9^ which may hinder rural dwellers from accessing the services provided by SBAs. Another reason for the disparity between urban and rural areas in the use of SBAs could be a result of the long travelling distances associated with health facilities in rural areas, especially in Papua New Guinea.^31,32^ In fact, as the study reveals, women who did not consider the distance to the health facility as being a big problem reported a higher use of SBAs compared with those women who did consider the distance to the health facility as being a big problem.

Utilisation of SBAs decreased with higher parity. This finding corroborates findings from studies in other countries.^9,33^ In this regard, women with high parity, with confidence, may feel that even in the absence of skilled providers they can still deliver their babies.^34^ This is more likely to be predominant among those women whose previous deliveries were supervised by unskilled providers. It is, therefore, necessary to educate such women that their status does not reduce the risk associated with unskilled assistance during delivery. Skilled delivery increased with more ANC visits, as reported elsewhere.^35–37^ This finding highlights the need for a renewal of efforts towards strengthening ANC programmes, so as to enhance skilled delivery in the country. Such efforts should, for example, include expanding coverage to enhance visits.

At the crude level, exposure to mass media was associated with higher odds of utilisation of skilled delivery services. Nonetheless, the results were attenuated after controlling for other covariates. In the adjusted model, women who were exposed to newspapers and watched television less than once a week had higher odds of SBA utilisation compared with those who were not exposed to newspapers or television at all. This is in line with a previous study by Fatema and Lariscy^38^ conducted in South Asia. Viswanath et al.^39^ postulated that mass media can influence attitudes towards healthy behaviour changes through community functions of information, tools, social control and healthy communication. Specifically, mass media can help ensure the update of health behaviours by consistently broadcasting programmes, public service announcements and advertisements that describe new drugs, novel treatments or risk factors. In Bangladesh, for example, the TV drama series *Ujan Ganger Naiya* showed maternal healthcare-related issues, including the advantages of delivery by SBAs, seeking ANC, adequate and prompt birth preparedness, postpartum check-ups, appropriate nutrition and essential newborn care, by entertaining audiences with stories set in a rural village.^38,40^ The media also offers health information, such as announcements of dates, times and places relating to when and where to seek healthcare. In south Asian countries, an educational media campaign about safe motherhood by the UNFPA included information about the symptoms of labour pain, as well as where and who would be best for delivering a baby.^41^

#### Strengths and limitations

At this point, we highlight some strengths and limitations of the study. In terms of strengths, given that the present study used nationally representative data, with its methodology validated, the findings of the study can be generalised to all women in the country. On the other hand, the data for the study are cross-sectional in nature, which makes it nearly impossible to ascertain causal inferences or temporal relationships between the studied variables. Again, the possibility of recall bias and social desirability among the study participants also presents another limitation to the study.

### Conclusion and policy implications

There is relatively low utilisation of skilled delivery services among women in Papua New Guinea. This study also revealed that individual-level factors such as wealth, employment status, educational level, parity and the number of ANC visits, as well as community-level factors such as access to healthcare facilities and place of residence, influence the utilisation of skilled delivery services in Papua New Guinea. In light of this, it is important for policies and programmes aimed at enhancing skilled delivery and reducing maternal mortality in the country to take these factors into consideration, so as to enhance the attainment of SGD 3.1. Such programmes should pay special attention to women who are resident in rural areas, as such women are more likely to use unskilled delivery services, due to their low socioeconomic status.

## Data Availability

The dataset can be accessed at https://dhsprogram.com/data/dataset/Papua-New-Guinea_Standard-DHS_2017.cfm?flag=0.
